# Adaptations in antagonist co-activation: Role in the repeated-bout effect

**DOI:** 10.1371/journal.pone.0189323

**Published:** 2017-12-07

**Authors:** Robert E. Hight, Travis W. Beck, Debra A. Bemben, Christopher D. Black

**Affiliations:** Department of Health and Exercise Science, University of Oklahoma, Norman, Oklahoma, United States of America; Cinvestav-IPN, MEXICO

## Abstract

Eccentric exercise results in an adaptation which attenuates muscle damage from subsequent exercise—termed the “repeated-bout effect (RBE).” **Purpose**: Study examined antagonist co-activation and motor-unit recruitment strategy, assessed via dEMG, concomitant to the RBE. **Methods**: Nine participants performed 5 sub-maximal isometric trapezoid (ramp-up, hold, ramp-down) contractions at force levels corresponding to 50% and 80% of maximal isometric strength (MVC). Surface EMG signals of the biceps brachii were decomposed into individual motor-unit action potential trains. The relationship between mean firing rate (MFR) of each motor-unit and its recruitment threshold (RT) was examined using linear regression. Eccentric exercise was then performed until biceps brachii MVC had decreased by ~40%. Surface EMG of the biceps and triceps were collected during eccentric exercise. MVC, range-of-motion (ROM), and delayed onset muscle soreness (DOMS) were measured 24-hours, 72-hours, and 1-week following eccentric exercise. Three weeks later all procedures were repeated. **Results**: Changes in MVC (-32±14% vs -25±10%; p = 0.034), ROM (-11% vs 6%; p = 0.01), and DOMS (31.0±19mm vs 19±12mm; p = 0.015) were attenuated following the second bout of exercise. Triceps EMG was reduced (16.8±9.5% vs. 12.6±7.2%; p = 0.03) during the second bout of eccentric exercise. The slope (-0.60±0.13 vs -0.70±0.18; p = 0.029) and *y*-intercept (46.5±8.3 vs 53.3±8.8; p = 0.020) of the MFR vs. RT relationship was altered during contractions at 80% of MVC prior to the second bout of eccentric exercise. No changes were observed at 50% of MVC. **Conclusion**: A reduction in antagonist co-activation during the second bout of eccentric exercise suggests less total force was required to move an identical external load. This finding is supported by the increased negative slope coefficient and an increased *y*-intercept of the linear relationship between RT and MFR.

## Introduction

Unaccustomed eccentric exercise often results in damage to individual sarcomeres—termed exercise-induced muscle damage (EIMD). Typical symptoms of EIMD include decreased strength, delayed-onset muscle soreness (DOMS), localized edema, decreased range-of-motion (ROM), and increased muscle-specific proteins such as creatine kinase (CK) in the blood (see [1] for review). Following EIMD, an adaptation occurs which attenuates the magnitude of EIMD and its associated symptoms following subsequent bouts of eccentric exercise [2–5]. This adaptation has been termed the “repeated-bout effect” (RBE) [2] and may last for 6 to 9 months [6]. While the exact mechanism(s) underlying the RBE are not fully resolved, two broad categories of adaptations have been hypothesized [7]: 1) peripheral changes within the muscle and 2) neural changes leading to alterations in motor-unit recruitment and activation.

Eccentric actions are thought to employ a unique recruitment strategy compared to concentric actions [8] whereby fewer motor-units may be utilized to generate a given force compared to concentric contractions and recruitment order may not follow the size principle [9]. These unique neural strategies likely play a role in the EIMD response to eccentric contraction as type II muscle fibers are thought to be more susceptible to EIMD [10] and heightened specific force (i.e. the force per active area of muscle) has also been shown to lead to EIMD [11–13]. As such, altered recruitment strategies whereby additional motor-units, particularly type I units, are recruited at any given force level could plausibly play a role in the RBE. Evidence from eccentric resistance training supports this idea. A greater increase in the ratio of surface EMG (sEMG) amplitude to eccentric strength has been shown compared to concentric training, potentially indicating greater motor-unit recruitment [14, 15]. An adaptation of this type would lower the specific force during eccentric contractions and limit mechanical stress placed upon individual fibers which could limit EIMD [11–13]. Additionally, a decreased sEMG median frequency has also been observed during a repeated bout of eccentric exercise [16–18], which has been suggested to indicate greater recruitment of slow twitch motor-units. However, changes in the sEMG signal, especially in the frequency domain, are affected by factors such as amplitude cancellation and changes in conduction velocity [19] and as such may not reflect changes in motor-unit behavior *per se*. Thus, sEMG is generally considered to represent a crude indicator of neural drive to skeletal muscle [20].

Greater insight into the behavior of individual motor-units requires the use of intramuscular EMG recordings and/or the decomposition of the surface EMG signal into its constituent motor-units. Using intramuscular recordings, increased synchronization of motor-unit firings [21] and decreased recruitment thresholds [22] have been observed 24-hours following an initial bout of damaging eccentric exercise. Using surface arrays an acute bout of eccentric exercise was shown to decrease conduction velocity [23–25], increase the slope and *y*-intercept of the mean firing rate (MFR) vs. recruitment threshold (RT) relationship [26], but not to effect common drive [27]. To date no studies have used a surface array to examine whether longer term (>24-hours) alterations in motor-unit behavior occur following a single bout of damaging eccentric exercise. Only one study [28] has assessed longer term (7 days after eccentric exercise) adaptations in motor-unit behavior using intramuscular recordings. In this study Dartnall et al. [28] found an increase in the synchronization of motor-unit firings, but no changes in RT 7 days after the initial bout of eccentric exercise. Increased synchronization has been suggested as a potential mechanism of the RBE [29]. Dartnall et al. [28] found attenuated EIMD following a repeated bout of eccentric exercise, but maximal isometric force was still reduced (~10%) 7 days following the initial bout—indicating the muscle may not have been fully recovered when the repeated bout was performed. As such it is unclear if the increased synchronization can be attributed to a lasting adaptation of motor-unit behavior or if it was a consequence of the muscle not being fully recovered from EIMD.

Given the paucity of data concerning motor-unit behavior and the RBE we sought to build upon the studies of Dartnall et al. [28] and Ye et al. [26] and use traditional sEMG as well as dEMG to examine changes in neural drive and motor-unit behavior prior to and during 2 bouts of damaging eccentric exercise separated by 3 weeks (to allow for full recovering of the muscle). Based upon previous studies it was hypothesized: 1) the second eccentric exercise bout would result in attenuated markers of EIMD [30], 2) sEMG median frequency would decline and sEMG amplitude (assessed via the root mean square) would increase during the second bout of eccentric exercise [16, 17] and 3) an increased linear slope coefficient and increased *y*-intercept of the MFR vs. RT relationship would be observed prior to the second bout of eccentric exercise [26]. A secondary aim was to examine whether changes in motor-unit behavior, if they occurred, were dependent upon contraction force. To that end the MFR vs. RT relationship was examined during contractions at two force levels—50% and 80% of MVC.

## Materials and methods

### Participants

Ten college-aged (6 men and 4 women) individuals who were physically active, but had not participated in heavy (≥70% of 1-RM) upper body resistance training for the previous 6 months were recruited for this study. One female participant was not able to perform the isometric contractions required for the decomposition of the surface EMG recording in the prescribed manner and her data were excluded from analysis leaving a sample of nine (6 men, 3 women) with a mean age of 24.7 ± 4.8 years, mean height of 176.4 ± 8.3 cm, and mean weight of 78.4 ± 14.6 kg. The University of Oklahoma Institutional Review Board approved the study and all participants provided written informed consent and completed routine medical screening, including an exertional rhabdomyolysis risk factor questionnaire, prior to testing. All testing was carried out in accordance with the approved guidelines from the IRB. Potential participants who reported a history of musculoskeletal injury, use of prescription pain or psychiatric medications (including medication for ADHD), or nutritional supplements were excluded from the study. Participants were instructed to refrain from resistance training and alterations in their diet or sleep habits over the course of the study and to refrain from consumption of NSAIDS or the use of other treatment modalities (ice, heat, massage) during the study and their compliance was confirmed prior to each testing session via self-report. A sample size of 9 was sufficient to detect a moderate (Cohen’s d of 0.49 SD) effect (change) in the mean of the slope coefficient and *y*-intercept of the RT vs. MFR relationship with a power of 0.80 and an alpha level of p<0.05 [31].

### Experimental overview

**Fig 1:**
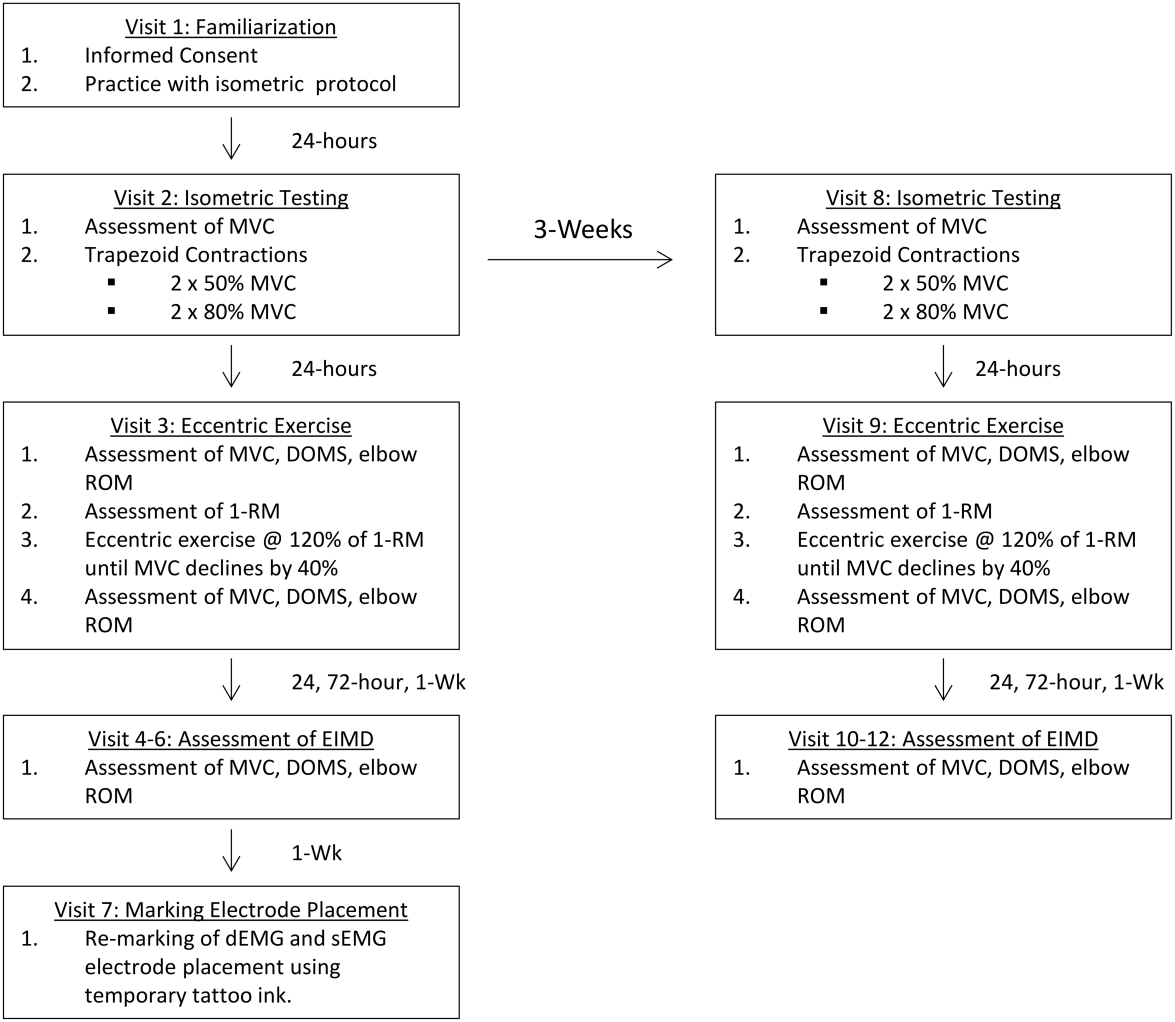
Overview of experimental design and procedures.

Twelve visits to the laboratory were required (see Fig 1). During the first visit, participants were familiarized with the experimental procedures including dEMG electrode placement, the testing of maximal voluntary strength (MVC), and performance of the trapezoid contractions used to determine motor-unit recruitment. Following familiarization, two experimental testing sessions, separated by 3 weeks were performed to assess motor-unit recruitment. During these visits, participants performed 2 MVCs followed by a series of submaximal isometric trapezoid contractions. Twenty-four hours following each motor-unit recruitment test, an eccentric exercise protocol (Bout 1 and Bout 2) was completed to induce EIMD. During the eccentric exercise surface EMG recording were made from the biceps brachii and the triceps brachii. Assessments of MVC, elbow ROM, and ratings of perceived muscle soreness, were performed 24-hours, 72-hours, and 1-week following each bout of eccentric exercise to assesses EIMD.

### dEMG recording

dEMG signals were recorded from the biceps brachii using a surface sensor array (Delsys, Inc., Natick, MA) that consisted of four pin electrodes arranged in a square with a fifth pin electrode located in the center of the square at a distance of 3.6 mm from each of the corner electrodes. This arrangement allows four bipolar surface EMG signals to be recorded simultaneously based on the pairwise differences between the pin electrodes [see Nawab et al. [32] for additional details regarding the dEMG sensor array]. Each of the four bipolar surface EMG signals were collected and amplified using a Bagnoli 16-channel system and the signals were sampled at 20 kHz and stored on a desktop PC for offline analysis.

Prior to electrode placement, the skin was shaved, abraded, and cleansed with 70% isopropyl alcohol. The dEMG sensor was then fixed over the belly of the biceps brachii muscle with medical tape and a ground electrode was placed over the acromion process of the non-dominant shoulder. When signal quality or decomposition accuracy from the original sampling site was poor, the dEMG sensor was moved to locations lateral and distal to or medial and proximal to the center of the muscle belly. Once a suitable site was located, it was marked with semi-permanent tattoo ink (Black Jagua; Earth Henna, Los Angeles, CA). The ink was reapplied as needed over the intervening 3 weeks of study participation to ensure similar dEMG sensor placement during the second assessment.

### MVC and trapezoid isometric contractions

Participants were seated on a preacher curl bench and positioned their arm on a rigid brace that secured the elbow at 90° of flexion. A padded cuff was fastened around the wrist and was connected to a force transducer (model SSM-AJ-500; Interface; Scottsdale, AZ) via a high-tension cable. The height of the preacher curl bench and distance from the force transducer were adjusted to ensure that each participant’s arm was positioned at 90°s of elbow flexion and that the line of pull from the cable was perpendicular to the force transducer. Following placement of the dEMG sensor and ground electrode, participants performed three submaximal isometric contractions (at ~50% of MVC) to warm up the elbow flexors and to allow for visual inspection of the dEMG signal-to-noise ratio to ensure proper signal acquisition. Two MVCs were then performed with three minutes of rest provided between contractions. Strong verbal encouragement and biofeedback of force output were provided to aid in participants providing a maximal effort. Following 5-minutes of rest, participants then performed 4 sub-maximal isometric contractions referred to as “trapezoid” contractions due to the fact that force was ramped up, held, and then ramped down in a manner that the force tracing resembled a trapezoid. During the trapezoid contraction, participants followed a force tracing on a computer monitor by linearly increasing force production (ramp-up) at a rate of 10% MVC per second to the prescribed target force (either 50% or 80% of MVC), held the force output steady, and then linearly decreased force production (ramp-down) from the target force level to 0% at a rate of 10% MVC per second. Target force levels were alternated (50, 80, 50, and 80% MVC) and each attempt was separated by 5 minutes of rest. The rate of force output was standardized at 10% of MVC/s and the length of the plateau phase decreased with an increase in target force levels (8 and 6 seconds for the 50%, and 80% contractions, respectively). This alternating protocol was employed to minimize effects of contraction order and to minimize the effects of muscular fatigue (which pilot testing indicated was likely if two 80% contractions were performed back-to-back) on motor-unit activity.

### Eccentric exercise protocol

Twenty-four hours following assessment of motor-unit recruitment, participants completed an exercise protocol designed to induce muscle damage consisting of unilateral eccentric contractions of the dominant elbow flexors. During the eccentric contractions, bipolar sEMG signals were recorded from the biceps brachii and triceps brachii muscles using a BioNomadix dual-channel wireless EMG system (Biopac, Goleta, CA). The skin was abraded and cleansed with 70% isopropyl alcohol and a pair of silver-silver chloride EMG electrodes (Biopac, Goleta, CA) were placed ~16 mm apart over the belly of the biceps brachii and the belly of the lateral head of the triceps brachii in accordance with SENIAM recommendations. A ground electrode was placed over the styloid process of the ulna and the wireless sEMG system was secured around the forearm with a velcro strap. A twin-axis electro-goniometer (Biopac, Goleta, CA) was attached with medical tape immediately superior and inferior to the lateral epicondyle of the humerus to provide information regarding the position of the elbow in order to allow the sEMG signal to be obtained from the same portion of the range-of-motion between bouts and among participants.

Unilateral, concentric 1-repetition maximal elbow flexor strength (1-RM) was assessed in the dominant arm. The weight was taken at the top of the movement by an investigator to prevent extraneous eccentric muscle actions that could potentially damage the muscle and confound the experimental protocol. Five minutes of rest were provided between each attempt and 1-RM was generally established within three to four trials. The heaviest weight successfully lifted was designated as the 1-RM. Participants then performed 2 MVCs with the elbow flexors and 2 with the elbow extensors. During each MVC, a 2 second average of the root mean square (RMS) amplitude of the sEMG signal corresponding to the plateau in force during these isometric contractions were recorded, averaged and then used to normalize sEMG recordings made during eccentric exercise. The eccentric exercise protocol consisted of performing three-second eccentric repetitions with a weight equal to 120% of their concentric 1-RM in sets of 10 until the participant’s MVC fell to 60% of their baseline value (i.e. a drop is force production of 40%) [33]. Five minutes of rest were provided between sets and a minimum of 3 sets of 10 were performed. MVC was re-assessed after each set. If MVC had not decreased to ~60% of baseline, an additional set of 10 repetitions was performed, and MVC was re-assessed. Exercise continued in this manner until the required decline in MVC was achieved. This protocol was used in an effort to provide a similar magnitude of damage to each participant as the damage response has been shown to be highly variable [34].

### Assessment of eimd

MVC, elbow ROM, and DOMS were assessed prior to (Pre), immediately after (iPost), 24-hours (24hr), 72-hours (72hr), and 1-week (1wk) following eccentric exercise. MVC was determined as described above. Elbow ROM was evaluated with a goniometer by determining resting elbow angle (the angle at the elbow as the arm hung relaxed at the side) and flexed elbow angle (the angle at the elbow as the joint was flexed until contact was made between the upper and lower arm) and calculating the difference between the two measures. DOMS was quantified using a visual analog scale (VAS). The scale consisted of a 100 mm line where “0” represented “no pain” and “100” represented “most intense pain imaginable.” Participants were asked to actively flex and extend the arm using a 15 lb dumbbell and mark their perceived level of soreness during the movement on the line.

### Signal processing and analysis

**Fig 2:**
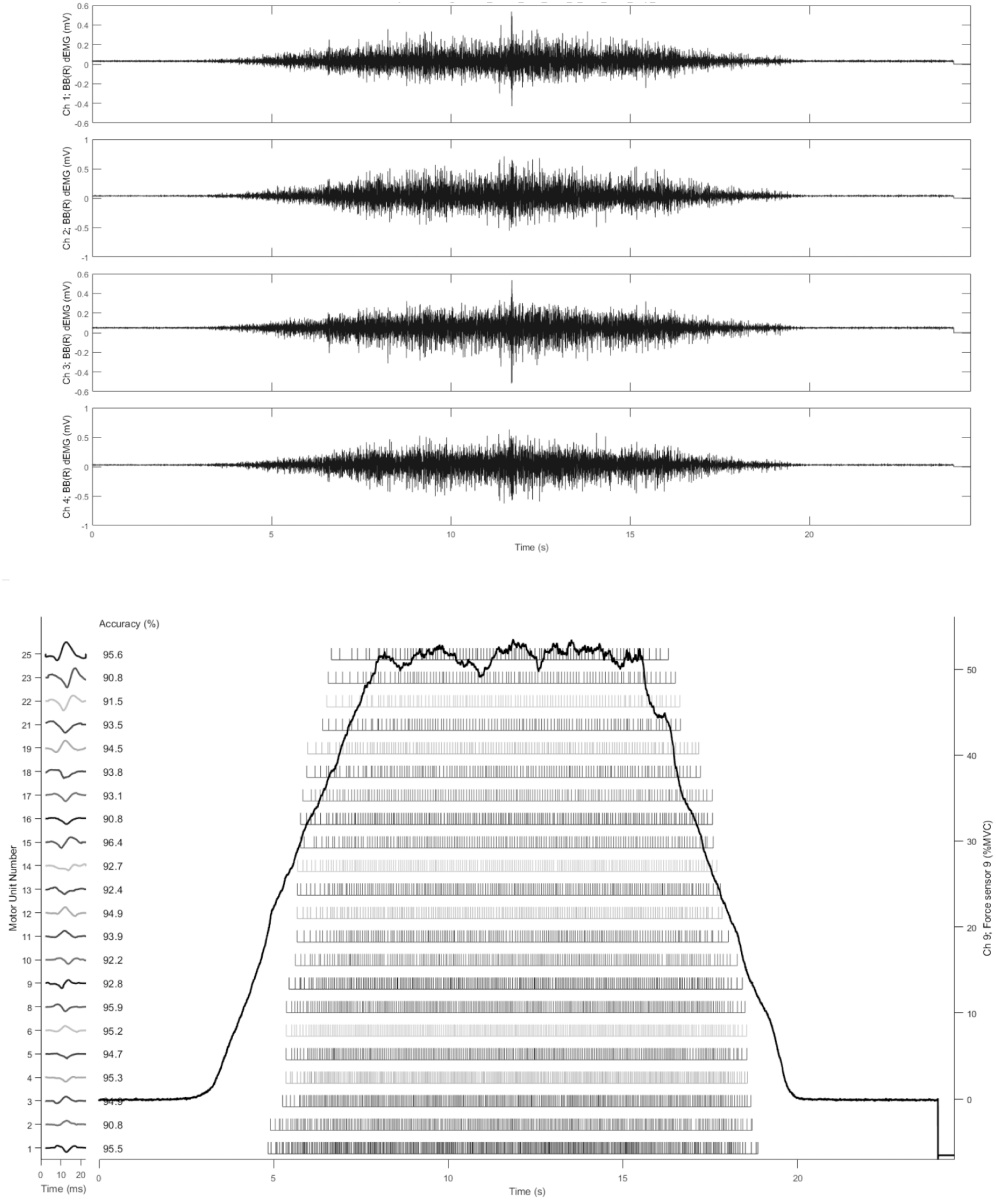
Example of raw EMG and individual firing incidences. Example of raw EMG signal collected from the 4 pin sensor during a contraction at 50% of MVC are shown in Panel A. Individual firings incidences of the 25 motor-units detected by the decomposition algorithm are shown in Panel B. Each bar represents the firing time of an action potential. The force tracing from the trapezoid contraction is shown by the overlaid solid line.

The dEMG signals were processed using EMGWorks v.4.1.7 (Delsys Inc., Natick, MA). Trapezoid contractions that met the following criteria were selected for decomposition: 1) a signal-to-noise ratio > 2.0, 2) baseline noise interference < 4.8 μV, and 3) and line interference < 1.0 (normalized power spectrum at 60 Hz). Contractions with sudden changes in the force trajectory (>10% MVC/s outside of the prescribed ramp-up, hold, and ramp-down protocol) were excluded from analysis. All analog EMG signals were low-pass filtered (cut-off frequency = 9500 Hz) and high-pass filtered (cut-off frequency = 20 Hz) at a sampling rate of 20,000 Hz. The filtered EMG signals from the four EMG channels were decomposed into their constituent motor unit action potential trains (MUAPTs) using the Precision Decomposition III (PD III) algorithm that was first described by De Luca et al. [35] and subsequently revised by Chang et al. [36] and Nawab et al [32]. This algorithm uses artificial intelligence techniques to separate superimposed action potentials from the EMG signals and assign it to an individual MUAPT belonging to a specific motor unit. The accuracy of the decomposed firing instances was examined using the Decompose-Synthesize-Decompose-Compare test described by Nawab et al. [32]. This method of accuracy assessment has clear advantages over other proposed methods of accuracy assessment [see Kline and De Luca [37] for review]. Only motor-units that were decomposed with >90% accuracy were included in subsequent analyses. A mean firing rate curve was calculated from the individual MUAPTs for each individual motor-unit. MFR of each motor-unit was then calculated detected from the plateau region of the force trajectory to limit potential changes in MFR due to changes in force production. The recruitment threshold for each motor unit was calculated as the relative force level (%MVC) when the first firing occurred. When two contractions with the same target force level met the signal quality criteria and had similar levels of decomposition accuracy, the contraction that yielded motor-units with the widest range of RTs was selected for use in further analysis. An example of the raw EMG signal from each of the 4 channels can be seen in Fig 2A. Fig 2B shows the individual firings, plotted as bars, of the 25 motor-units detected by the algorithm during a contraction at 50% of MVC with the corresponding trapezoid force tracing overlaid.

sEMG recordings collected during the eccentric contractions were analyzed using Biopac AcqKnowledge software (version 4.4). The raw EMG signals from the biceps brachii and triceps brachii were collected at a sampling rate of 2000 Hz and band-pass filtered at with high and low cutoff frequencies of 10 Hz and 500 Hz respectively. The RMS was calculated using an average with a time constant of 30 ms. Median frequency (MF) of the power density spectrum was calculated via fast Fourier transform computed in 500 ms epochs. Each eccentric contraction took approximately 2.5 to 3.0 seconds to complete. As such the mean RMS and MF from both muscle groups were determined from the middle two seconds of each contraction (as determined from the electro-goniometer) in order to minimize between bout and among participant variation. Mean RMS values from each eccentric contraction were normalized and expressed as a percentage of the mean RMS values from biceps brachii and triceps brachii MVC, respectively. RMS and MF from the initial 2, middle 2, and final 2 contractions of each set of eccentric contractions were averaged and reported for the first set, middle set, and final set of 10 eccentric contractions.

### Statistical analyses

MVC, ROM, and DOMS were analyzed with a 2 (bout) by 5 (time) completely within participant analysis of variance (ANOVA) to determine the magnitude of EIMD. EMG RMS and MF were analyzed using a 2 (bout) by 9 (time) completely within participant ANOVA. If significant interactions were found, they were followed by 1-way ANOVA’s for time and planned contrasts to examine differences in mean values at each time point compared to values at PRE. Planned contrasts were also performed to examine differences between bouts at each time point. Main effects were only interpreted in the absence of a significant interaction. If significant main effects were found, follow-up testing of main comparisons was performed using the Fisher’s LSD model as the study was designed and powered to make these comparisons. Mean values for the linear slope coefficients and *y*-intercepts of the MFR vs. RT relationship at 50% and 80% of MVC were analyzed using dependent t-tests. Individual motor-units were placed in groups based upon their RT and separated into “bins” corresponding to 10% increments in RT. The mean of the MFR of bin of motor units was then calculated and compared between bouts with a dependent t-test. Assumptions of sphericity were determined using Mauchly’s test and the Greenhouse-Geisser correction was applied if sphericity was violated. All statistical tests were performed with SPSS version 19 with alpha set *a priori* at 0.05. Effect sizes were calculated as a Cohen’s d statistic, as the difference in means divided by the pooled standard deviation of the means. As a general guideline, effects of ~0.20 SD are judged to be small, ~0.50 SD are judged to be moderate, and ≥ 0.80 SD are judged to be large.

## Results

### Assessment of eimd

**Fig 3:**
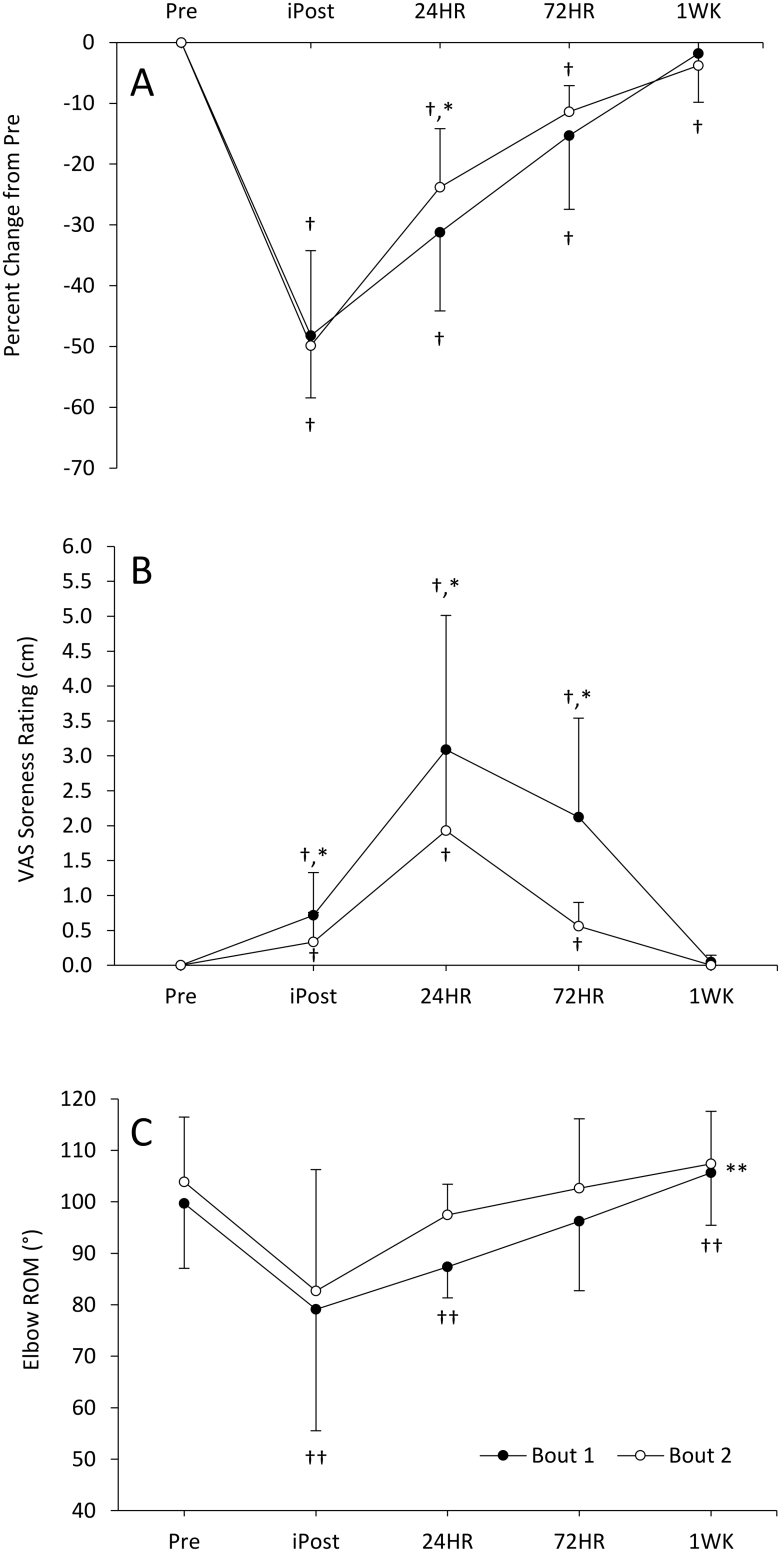
Assessments of muscle damage. Change (as a percentage of “pre”) in isometric strength of the elbow flexors (Panel A), ratings of muscle soreness in cm (Panel B), and elbow range-of-motion (Panel C) over one week following Bout 1 and Bout 2 (separated by 3 weeks) of eccentric exercise. † indicates a significant difference from Pre. * indicates a significant difference between bouts. ** indicates a main effect for bout. †† indicates a significant difference from Pre (main comparison). Values are mean ± SD.

Baseline MVC assessed prior to each motor-unit testing session (72.7 ± 20.5 Nm vs. 72.6 ± 23.7 Nm), and prior to (Pre) each eccentric exercise bout (70.2 ± 22.1 Nm vs. 72.1 ± 22.9 Nm for Bout 1 and Bout 2, respectively; p = 0.22) were similar and reliable with an intra-class correlation coefficient [ICC (1,3): two-way mixed, single measure model] value of 0.98. Panel A of Fig 3 displays the changes in MVC as a percent change from Pre over the 7 days following the eccentric exercise protocol. There was a significant bout by time interaction, (p = 0.031) and the 1-way ANOVA’s for time were significant for both Bout 1(p < 0.001) and Bout 2 (p < 0.001). During Bout 1 strength was significantly reduced compared to Pre values immediately post exercise (p = 0.00006), 24hr post (p = 0.00003), and 72hr post (p = 0.0005), but not at 1wk post (p = 0.75). During Bout 2, strength was reduced immediately post (p = 0.0007), 24hr post (p = 0.00003), 72hr post (p = 0.00002), and 1wk post (p = 0.009). When comparisons were made between bouts, no differences were observed immediately post (p = 0.61; d = -0.12), 72hr (p = 0.38; d = 0.48), and 1wk post (p = 0.62; d = -0.31)). However, strength was reduced to a greater extent at 24hr post (p = 0.03; d = 0.66) in Bout 1 compared to Bout 2.

Ratings of muscle soreness are displayed in Panel B of Fig 3. There was a significant bout by time interaction (p = 0.015). The 1-way ANOVA’s for time were significant for both Bout 1 (p < 0.001) and Bout 2 (p = 0.001). Ratings of soreness were elevated in Bout 1 immediately post eccentric exercise (p = 0.008), persisted 24hr (p = 0.001) and 72hr (p = 0.002) post and had returned to Pre values by 1wk post (p = 0.22). A similar pattern was observed during Bout 2 with soreness being elevated immediately post (p = 0.046), 24hr (p = 0.0009), and 72hr post (p = 0.001) and returning to Pre values 1wk post (p = 0.99) eccentric exercise. When compared between bouts, rating of soreness were significantly reduced immediately post (p = 0.04; d = -0.74), 24hr (p = 0.03; d = -0.76), and 72hr (p = 0.015; d = -1.78) post eccentric exercise in Bout 2 compared to Bout 1. No difference was observed at 1wk post (p = 0.22; d = -0.88).

Panel C of Fig 3 displays measurements of elbow ROM. There was not a significant bout by time interaction (p = 0.28). There was significant main effects for time (p < 0.001) with ROM being significantly reduced immediately post eccentric exercise (p = 0.002) and 24hr post (p = 0.002). Values did not differ from Pre at 72hr post exercise (p = 0.35) and ROM was increased compared to Pre at the 1wk post time point (p = 0.013). A main effect for bout was observed (p = 0.01) with values from Bout 1 being significantly reduced compared to Bout 2.

### sEMG amplitude and median frequency

**Fig 4:**
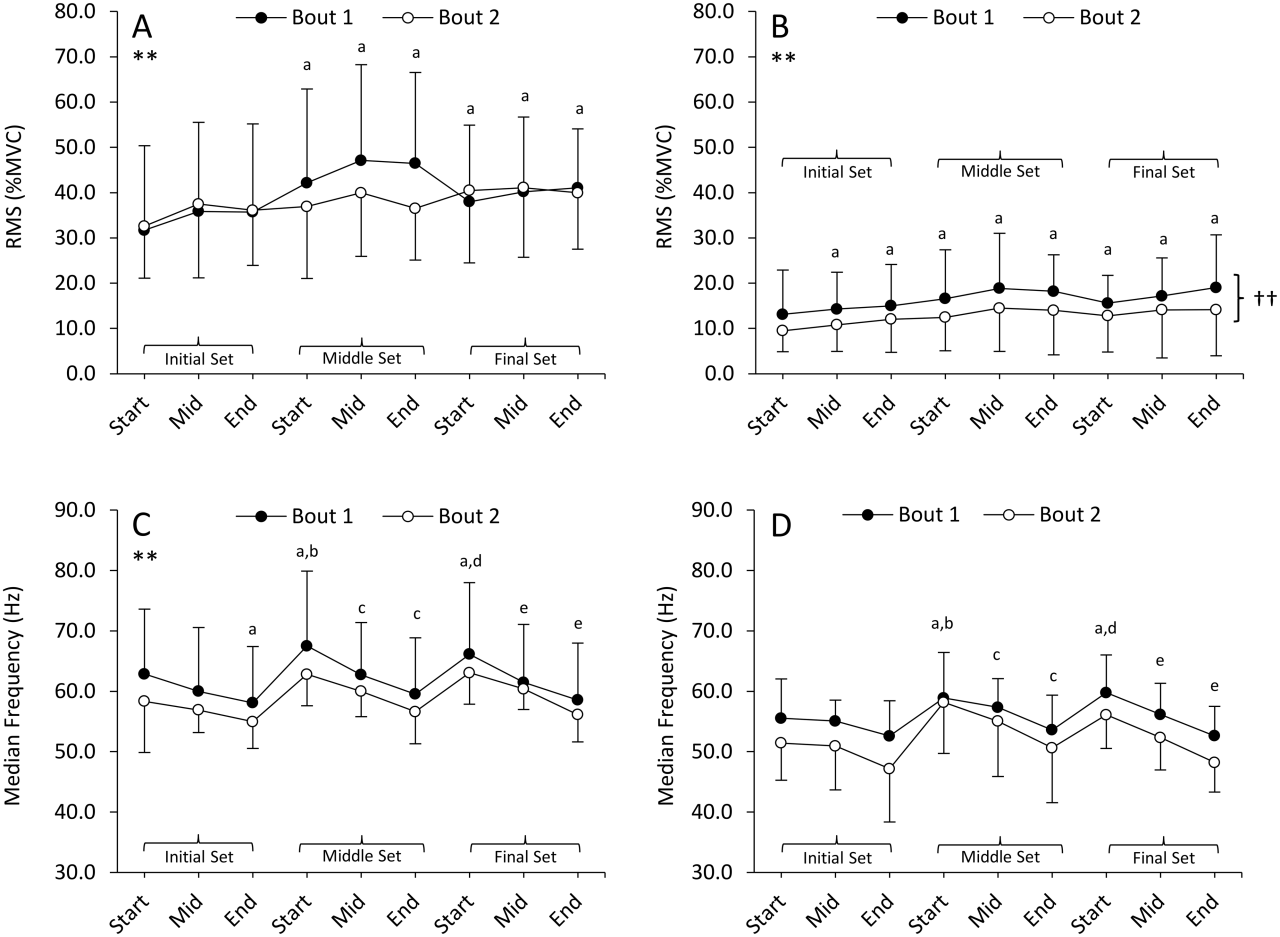
Surface EMG recording from the bicpeps and triceps brachii. Mean RMS amplitude in the biceps brachii (Panel A) and triceps brachii (Panel B) as well as median frequency in the biceps brachii (Panel C) and tricep brachii (Panel D) and during eccentric exercise. ** indicates a main effect for time. ^a^ indicates a significant difference “start” of the initial set; ^b^ significant difference from “end” of initial set; ^c^ significant difference from “start” of the middle set; ^d^ significant difference from “end” of the middle set; ^e^ significant difference from “start” of final set. †† indicates a significant main effect for bout. Values are mean ± SD.

There was no significant bout by time interaction for biceps RMS (p = 0.24), the bouts did not differ (p = 0.69 for main effect), but a significant main effect for time (Fig 4A; p = 0.04) was observed with contractions during the middle and final set demonstrating an increased RMS compared to the initial contractions in the initial set (p ≤ 0.05). For RMS from the triceps, the bout by time interaction was not significant (p = 0.69), but significant main effects found for bout (p = 0.027), with Bout 2 demonstrating reduced RMS compared to Bout 1, and time (p = 0.008), with RMS increasing across all contractions compared to the initial contractions in the initial set (p ≤ 0.02; Fig 4B). Median frequency of power spectrum did not differ between bouts for the biceps (p = 0.65 for interaction, and p = 0.24 for main effect of bout). A significant main effect for time (p = 0.001) was found with MF generally falling within a set (p ≤ 0.03), and then recovering at the beginning of the next set (Fig 4C). There was no interaction between bout and time for MF from the triceps (p = 0.65), but main effects for both bout (p = 0.027) and time were found (p < 0.001). MF did not change over the initial set (Fig 4D; p ≥ 0.05), but increased by the beginning of the middle set (p = 0.012), declined as contractions progressed in the middle set (p ≤ 0.032), again increased at the beginning of the final set (p = 0.016) and progressively declined over the final set (p ≤ 0.009).

### The relationship between recruitment threshold and mean firing rate

**Fig 5:**
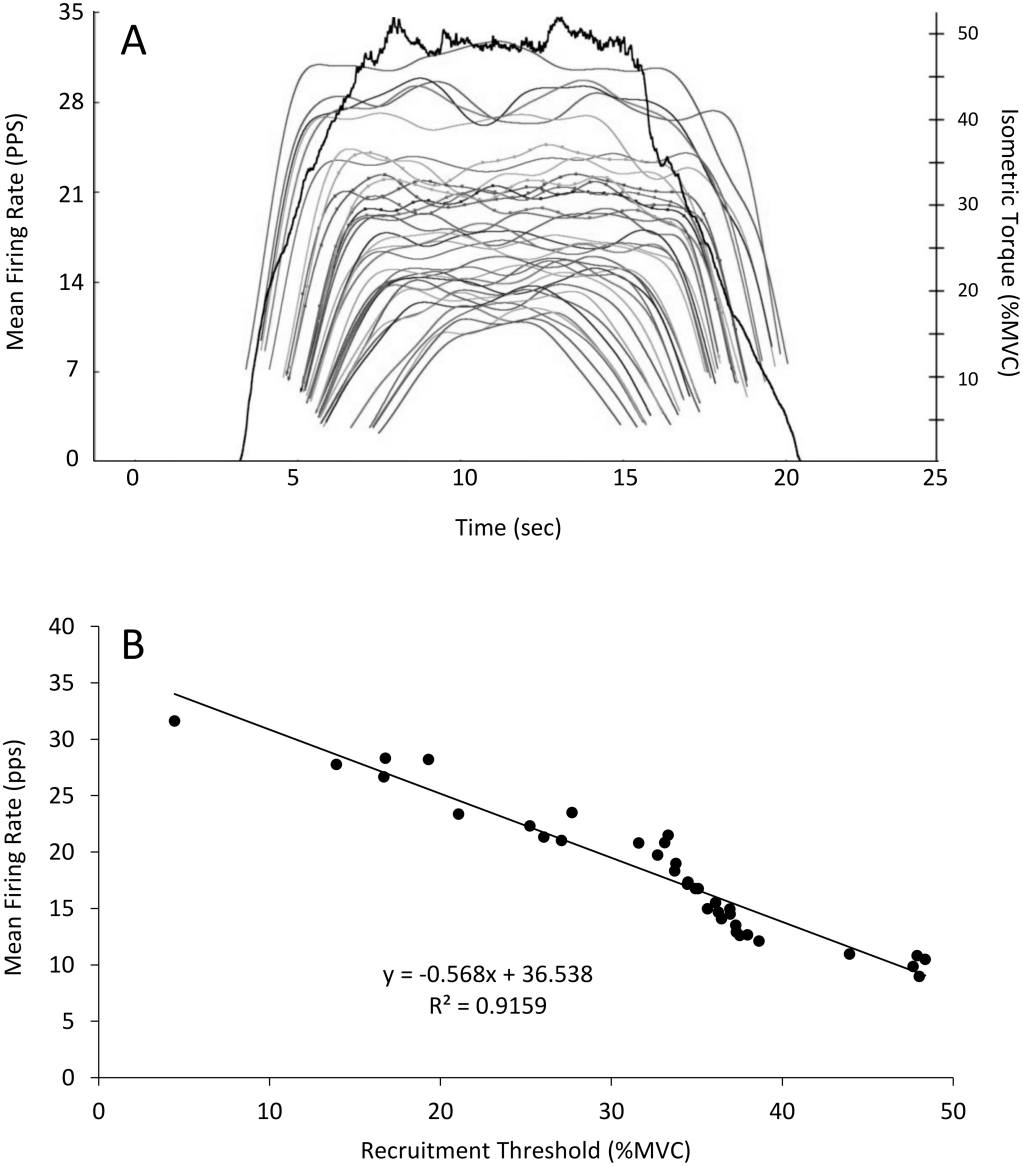
Onion skin plot of firing rates and recruitment thresholds for individual motor units. Mean firing rate plot of the biceps brachii (Panel A) or “onion skin” plot for a single participant prior to Bout 1 of eccentric exercise during a contraction at 50% of MVC. The solid black line shows torque production and the remaining curves represent individual motor units and their average firing rates over time. Panel B shows the linear relationship between recruitment threshold and mean firing rate for each identified motor unit.

**Fig 6:**
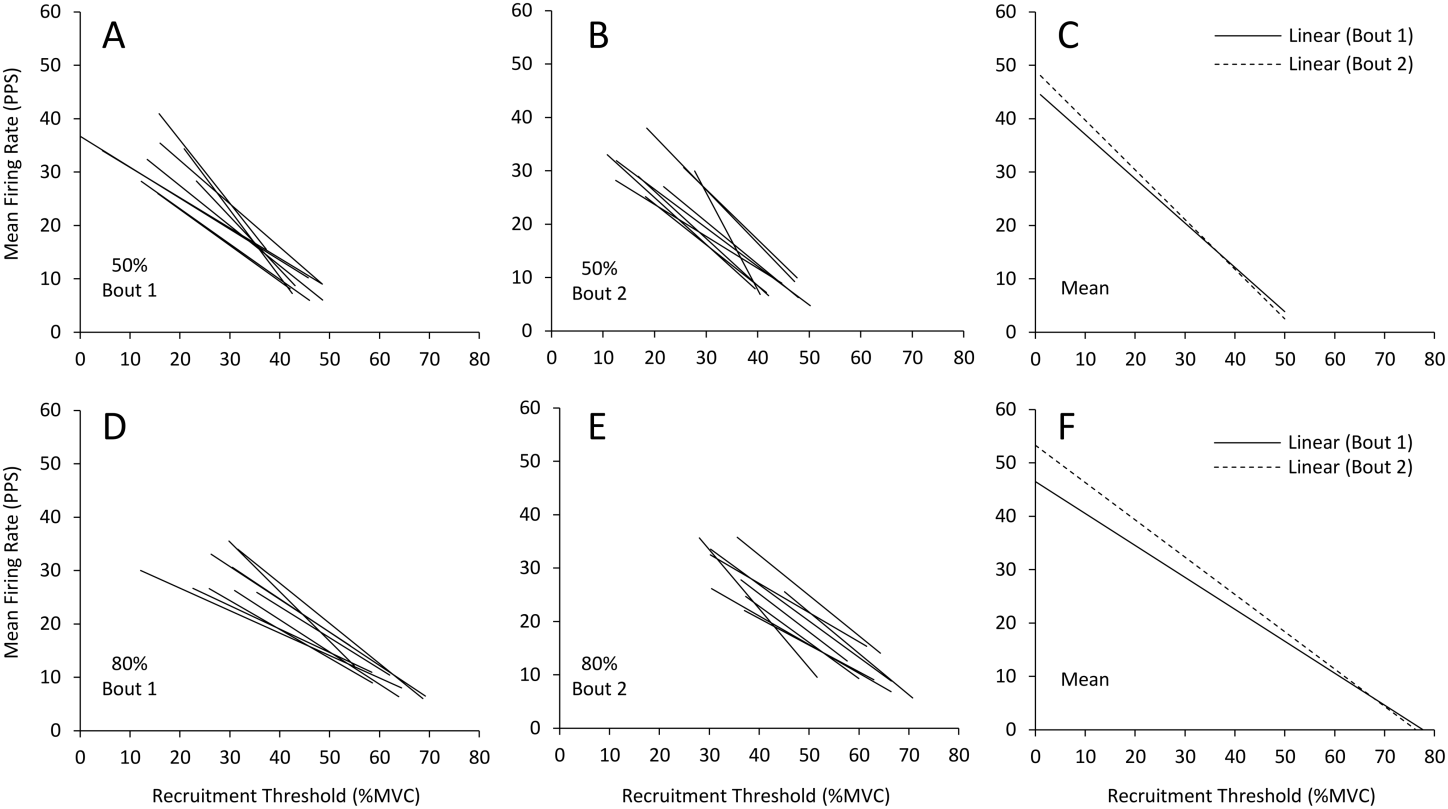
Relationship between recruitment threshold and firing rate for each participant. Linear regressions between recruitment threshold and mean firing rate of the biceps brachii for each participant during contractions at 50% (A; Bout 1 and B; Bout 2) and 80% (D; Bout 1 and E; Bout 2) of MVC. Panel C and F show the mean relationship across all participants for Bout 1 (solid lines) and Bout 2 (dashed lines) at 50% and 80% of MVC, respectively.

**Fig 7:**
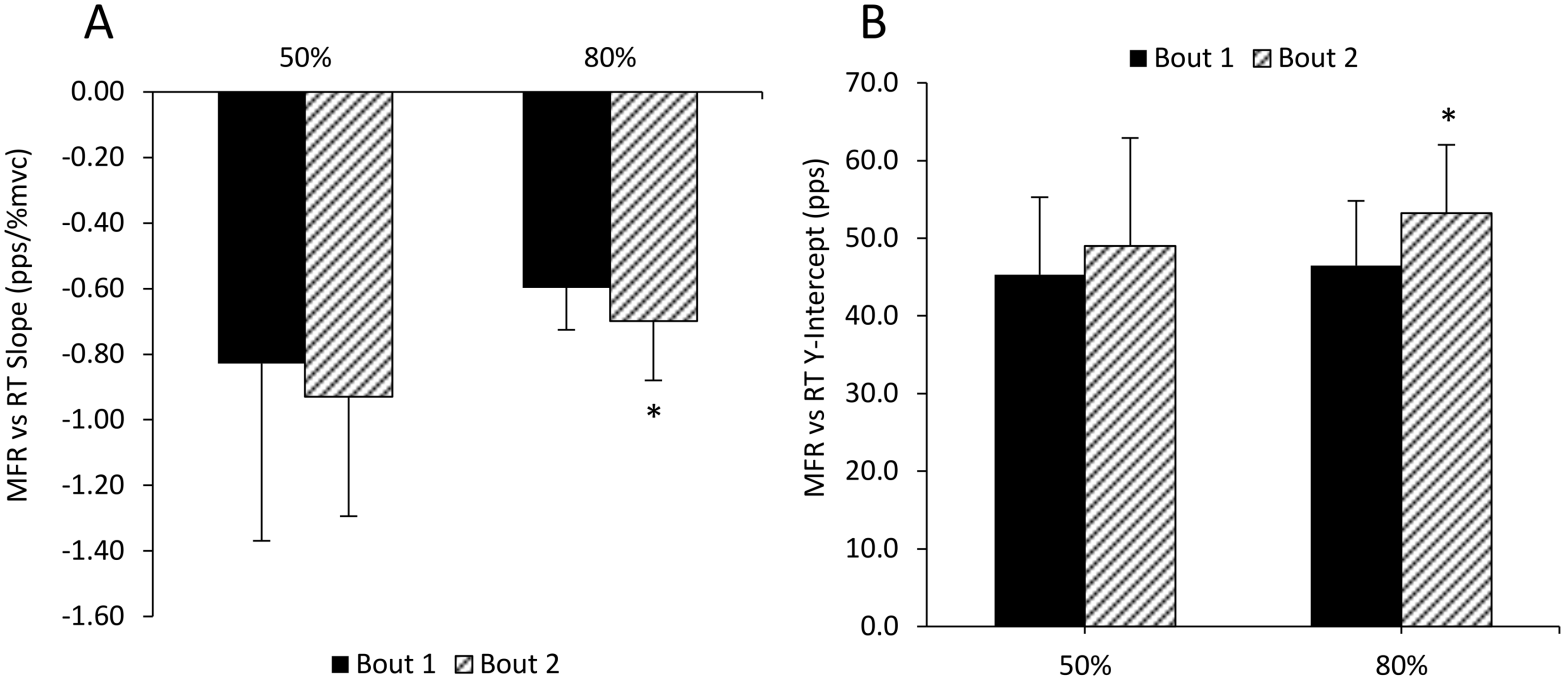
Change in slope and y-intercept of the firing rate vs. recruitment threshold relationship. Mean firing rate versus recruitment threshold linear slope coefficients (Panel A) and mean firing rate versus recruitment threshold *y*-intercept (Panel B) of the biceps brachii prior to Bout 1 and Bout 2 of eccentric exercise. Values are means ± SD. *indicates a significant difference between bouts.

**Fig 8:**
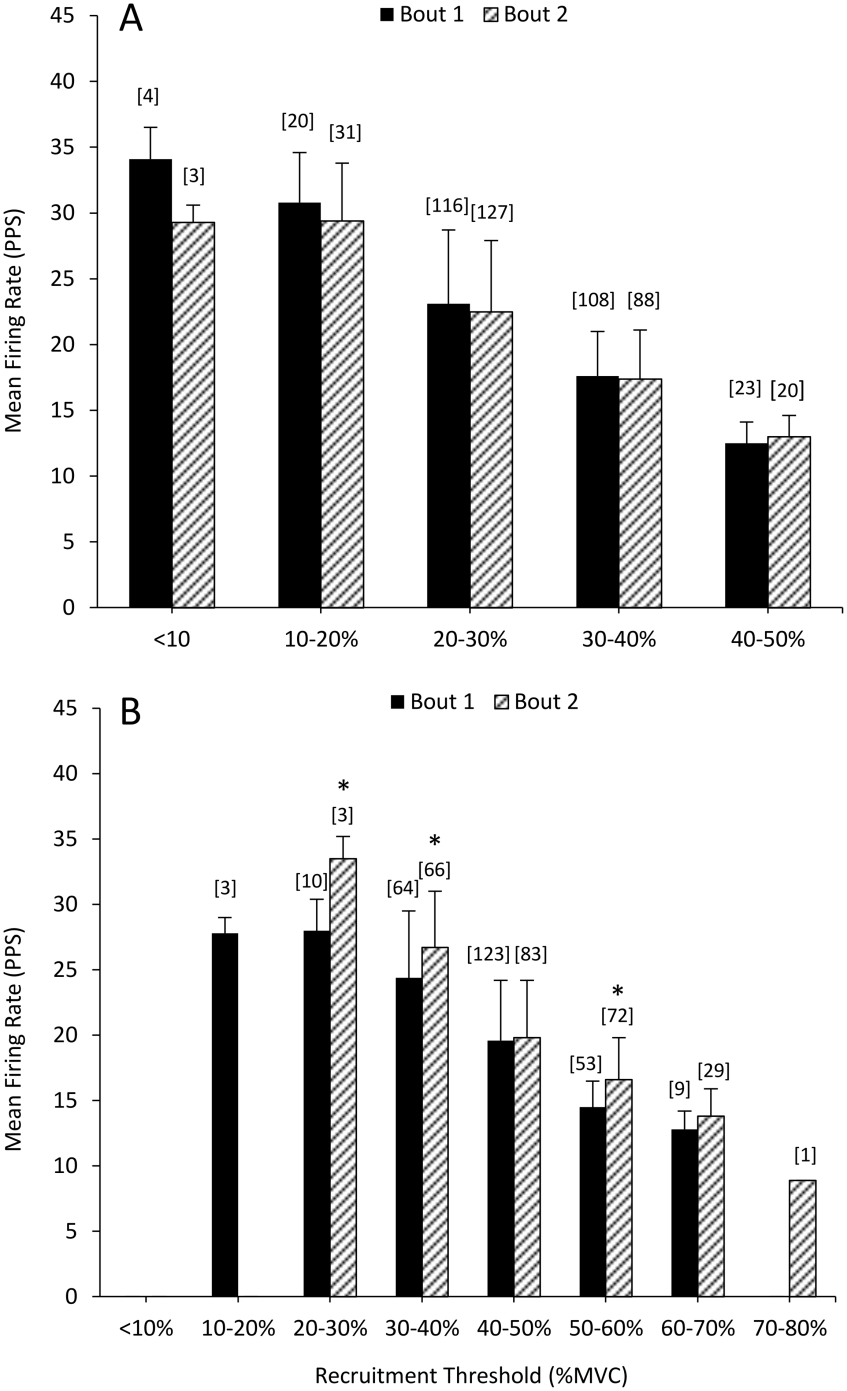
Firing rates for individual motor units grouped based upon recruitment threshold. Mean firing rate for individual motor-units of the biceps brachii separated into groups or bins corresponding to 10% of MVC. Panel A are data from contractions performed at 50% of MVC, Panel B are data from 80% of MVC. Number in brackets above each bar indicate the number of motor-units in each bin. *indicates a significnat difference between bout 1 and bout 2 (p < 0.05). Values are means ± SD.

Fig 5A shows an example “onion skin” plot demonstrating individual motor units of the biceps brachii during a contraction at 50% MVC and the corresponding linear plot of RT vs. MFR for each detected motor unit is shown in Fig 5B. Two hundred seventy nine (Bout 1) and 278 (Bout 2) total motor units were detected during contractions at 50% of MVC with 271 (Bout 1) and 269 (Bout 2) accurately detected and decomposed (e.g. ≥ 90% accuracy with the reconstruct-and-test method) yielding an average of 30.1 ± 6.0 and 29.9 ± 6.5 units per participant. During contractions at 80% of MVC 277 and 261 motor units were detected during Bout 1 and Bout 2, respectively with 262 and 250 accurately decomposed; 29.1 ± 7.7 and 27.8 ± 5.5 motor units per participant. The individual linear regressions calculated for each participant during contractions at 50% and 80% of MVC prior to Bout 1 and Bout 2 are shown in Fig 6. No changes were observed in the mean linear slope coefficient (Fig 6; p = 0.53; d = -0.36) or the *y*-intercept during contractions at 50% (p = 0.58; d = 0.25) between Bout 1 and Bout 2. There was a significant decrease (p = 0.02; d = -0.66) in the linear slope coefficient (i.e. the slope became steeper) and in increase in *y*-intercept (p = 0.02; d = 0.80) from Bout 1 to Bout 2 at 80% of MVC (Fig 7). Ther mean firing rate of indiviudal motor-units grouped into bins corresponding to 10% increments of MVC (e.g. groups from 0–10%, 10–20%, 20–30% of MVC, etc.) are shown in Fig 8. No differences were observed in the mean MFR between Bout 1 and Bout 2 in any recruitment threshold bin during contractions at 50% of MVC (p ≥ 0.22 for each). However, at 80% of MVC mean MFR was increased prior to Bout 2 compared to Bout 1 for motor-units with recruitment thresholds ranging from 20–30% (p = 0.004), 30–40% (p = 0.006), and 50–60% (p = 0.00004) of MVC.

## Discussion

A clear RBE was observed in the present study with all markers of EIMD (MVC, ROM and DOMS) being attenuated following Bout 2 of eccentric exercise. Our findings followed a similar time-course and were of a similar magnitude as previously reported [2, 3, 5, 17, 18, 38, 39]. Our primary novel findings were: 1) a reduction in elbow extensor co-activation during the second eccentric exercise bout; 2) an increased negative slope and an increased *y*-intercept of the RT vs MFR relationship assessed prior to Bout 2 compared Bout 1, during contractions performed at 80% of MVC; and 3) an increase in the mean firing rate of motor-units recruited between 20%-40% and 50%-60% of MVC during contraction at 80% of MVC prior to the second eccentric exercise bout.

To our knowledge no previous study of the RBE has examined potential changes in antagonist co-activation during eccentric exercise. Therefore, our finding of reduced triceps co-activation represents and interesting and here-to-fore unexamined change in neural strategy that would underlie the RBE. With decreased triceps co-activation, a greater portion of elbow flexor force would be applied to the external load—rather than being used to overcome the stabilizing force of the elbow extensors. The external load lifted was unchanged from Bout 1 to Bout 2. Consequently, a decrease in co-activation would result in less total force being generated by the flexors to move the external load. Since flexor RMS amplitude was unchanged from Bout 1 to Bout 2, generating a smaller force would result in a decreased force per active area of contracting muscle which has been shown to decrease EIMD [11–13]. As such, our novel fining of decreased co-activation represents a plausible mechanism by which a change in neural strategy could result in the RBE.

In an effort to provide additional data regarding motor-unit behavior and the RBE, dEMG was employed in the present study. Previous studies [40–42] have described the inverse relationship between motor-unit firing rate and recruitment threshold—often termed the “onion skin” relationship. When this pattern is observed, motor-units with a lower recruitment threshold fire at higher rates than motor-units recruited later (i.e. high-threshold) at any given absolute force. De Luca and Hostage [43] used dEMG and linear regression analysis to examine changes in the slope of the RT vs. MFR relationship as force was increased from 20%-100% of MVC across several muscles. As larger, faster motor-units were recruited and driven at higher firing rates, the slope of RT vs. MFR relationship decreased (i.e. became flatter). This relationship was robust across muscles suggesting a control scheme whereby the RT of a motor-neuron determines its firing characteristics based upon the level of excitation [43]. The slope coefficients and *y*-intercept of the RT vs. MFR relationship have since been used to study the effects of fatigue [26, 44], resistance training [45], and fiber type composition [46] on this control strategy. Similar to previous work [43, 46] in the present study increasing force production from 50% of MVC to 80% of MVC resulted in an increase in the linear slope coefficient (i.e. it became less negative and less steep). This change is likely due the recruitment of additional higher-threshold motor-units, which fire at lower rates, as force production is increased.

In the present study no changes in the linear RT vs. MFR relationship were observed prior to the second eccentric exercise bout during contractions at a moderate force level of 50% of MVC. However, at 80% of MVC the linear slope coefficient became more negative (decreased; became more steep) and the *y*-intercept increased prior to Bout 2 compared to Bout 1. Participants’ MVC had fully recovered during the 3-week period between Bout 1 and Bout 2, therefore 80% of MVC represented a similar absolute force. The change in slope and *y*-intercept is consistent with a reduced level of overall excitation being required to generate an identical target force. Little is known regarding long-term changes in spinal and supra-spinal centers following eccentric exercise. Seven weeks of eccentric training was found to enhance voluntary drive from supra-spinal centers but did not alter spinal excitability [47]. Whether a single bout of eccentric exercise is sufficient to evoke similar changes or whether changes would persist for 3 weeks with no further training remain unclear. Recent findings have shown acute (up to 48-hrs) deficits in motor cortex output in the days immediately following EIMD, but cortiospinal excitability recovered and was unchanged with the RBE [39]. Our findings of reduced triceps co-activation during eccentric exercise provide another adaptation that could plausibly explain the shift in the RT vs. MFR relationship. With less co-activation to overcome, the flexors would have to generate less total force (and therefore require less total excitation) to produce the same absolute external force—thus shifting the RT vs. MFR relationship in a manner consistent with a lower force contraction.

A potential consequence of reduced total force generation by the flexors, due to reduced triceps co-activation, would be to reduce the relative stress applied to high-threshold, type II motor units. The steeper slope and increased *y*-intercept along with the finding of increased MFR of motor-units recruited between 20%-40% and 50%-60% of MVC are consistent with lower-threshold, presumably slow-twitch motor-units contributing a greater portion of the total force generated by the muscle. Slow twitch muscle fibers are thought to be less susceptible to EIMD than their fast counterparts [10] and a shift in recruitment patterns to generate and distribute a greater proportion of force among slow fibers has been suggested [7] to underlie the RBE. We propose that rather than a change in the overall control strategy and a “shift” in recruitment away from type II motor-units that reduced co-activation lowers the required agonist force, the same recruitment scheme is applied, and fewer high-threshold units are needed to generate the required force.

This study has several important experimental considerations. Recent advancements in surface EMG decomposition techniques have greatly improved the ability to study motor-unit recruitment strategies and offer certain advantages (e.g. the ability to assess recruitment during higher force contractions) over the use of intramuscular recordings [32, 35, 36, 48]. However, it is important to recognize the limitations of the dEMG technique employed in this study. In order to accurately decompose the recorded signals, the recordings were obtained from ramp and hold isometric contractions rather than during the eccentric contractions that resulted in EIMD. A recent study has demonstrated similar motor-unit behavior during ramp and hold isometric contractions and dynamic contractions using the dEMG technique employed in this study [49]. However, until further studies are conducted the question of whether dEMG derived motor-unit behavior during isometric contraction is truly indicative of behavior during eccentric contractions remains somewhat unresolved. Another consideration was the range of contraction forces used. Pilot testing revealed using dEMG at force levels over 80% of MVC yielded inconsistent, and less accurate motor-unit decomposition. Consequently, maximal effort isometric contractions, which might more closely approximate the recruitment strategies employed during maximal effort eccentric contractions, were not used. However, previous findings [42] have found near maximal motor-unit recruitment at force levels approximating 85% of MVC—suggesting a near maximal level of motor-unit recruitment was likely even though force was only 80% of MVC. Finally, the validity of the decompose-synthesize-decompose-compare test [32] for the assessment of the accuracy of the decomposition algorithm has been questioned [50, 51]. While important questions have been raised, multiple validation protocols such as a direct test for bias [52], a two-source validation test [32], and a two-sensor validation test [35] have all demonstrated decomposition accuracy of ≥90%. Additionally, researchers independent of the manufacturer have also demonstrated high levels of accuracy for the decomposition of MUATP’s [53–56]. For a more in-depth review of the discussion surrounding the validity of the decomposition please see the comments by *De Luca* [57, 58].

In conclusion the primary novel finding of the present study was a reduction in antagonist co-activation during a second bout of eccentric exercise that results in less EIMD compared to an initial bout performed 3 weeks prior. Suggesting less total agonist force was required to move an identical external load. dEMG was also employed to examine motor-unit behavior prior to each bout of eccentric exercise. An increased negative slope coefficient and an increased *y*-intercept of the linear relationship between RT and MFR was observed—consistent with a lowered total force requirement. The present study provides further evidence a single bout of damaging eccentric exercise may provide a long-lasting change in neural recruitment strategy that functions to limit damage during subsequent bouts of eccentric exercise.

## Supporting information

S1 Dataset Dataset for all individual motor unit firings. (XLSX)

S2 Dataset Dataset for assessment of EIMD and surface EMG. (XLSX)

S3 Dataset Dataset for slope and y-intercepts of dEMG motor unit firings. (XLSX)
